# Identification of a *RAD51B* enhancer variant for susceptibility and progression to glioma

**DOI:** 10.1186/s12935-023-03100-8

**Published:** 2023-10-19

**Authors:** Liming Huang, Wenshen Xu, Danfang Yan, Xi Shi, Shu Zhang, Meiqin Chen, Lian Dai

**Affiliations:** 1 Department of Oncology, The Affiliated People’s Hospital, Fujian University of Traditional Chinese Medicine, #602 Bayiqizhong Road, Fuzhou, 350004 China; 2 Department of Laboratory Medicine, The First Affiliated Hospital, Fujian Medical University, Fuzhou, 350005 China; 3 Fujian Key Laboratory of Laboratory Medicine, The First Affiliated Hospital, Fujian Medical University, Fuzhou, 350005 China; 4 Department of Radiation Oncology, The First Affiliated Hospital, College of Medicine, Zhejiang University, Hangzhou, 310003 China; 5 Department of Medical Oncology, The First Affiliated Hospital, Fujian Medical University, Fuzhou, 350005 China; 6 Department of Medicine, The Third Affiliated People’s Hospital, Fujian University of Traditional Chinese Medicine, #363 Guobin Road, Fuzhou, 350108 China

**Keywords:** Glioma, *RAD51B*, Enhancer, Genetic variation, Susceptibility, Progression

## Abstract

**Background:**

RAD51B plays a significant role in homologous recombination-mediated repair of DNA double-strand breaks. Many enhancer variants are involved in cancer development and progression. However, the significance of enhancer variants of *RAD51B* in glioma susceptibility and progression remains unclear.

**Methods:**

A case–control study consisting of 1056 individuals was conducted to evaluate the associations of enhancer variants of *RAD51B* with glioma susceptibility and progression. Sequenom MassARRAY technology was used for genotyping. The function of enhancer variants was explored by biochemical assays.

**Results:**

A significantly decreased risk of glioma was associated with rs6573816 GC genotype compared with rs6573816 GG genotype (OR = 0.66, 95% CI 0.45–0.97; *P* = 0.034). Multivariable Cox regression revealed that rs6573816 was significantly associated with glioma progression in a sex-dependent manner. Worse PFS was found in the male patients with high grade glioma carrying rs6573816 GC or CC genotype (HR = 2.28, 95% CI 1.14–4.57; *P* = 0.020). The rs6573816 C allele repressed enhancer activity by affecting transcription factor POU2F1 binding, which resulted in lower expression of *RAD51B*. Remarkably attenuated expression of *RAD51B* was observed following *POU2F1* knockdown. Consistently, positive correlation between the expression of *POU2F1* and *RAD51B* was found in lymphoblastic cells and glioma tissues.

**Conclusions:**

These results indicate that an enhancer variant of *RAD51B* rs6573816 influences enhancer activity by changing a POU2F1 binding site and confers susceptibility and progression to glioma.

**Supplementary Information:**

The online version contains supplementary material available at 10.1186/s12935-023-03100-8.

## Background

Glioma is the most common form of primary malignant intracranial tumors. Numerous epidemiologic studies have revealed some genetic and environmental risk factors for glioma. Most noteworthy, ionizing radiation has been firmly established as an unequivocal environmental risk factor for glioma [1]. Ionizing radiation can induce various types of DNA damage, such as DNA single- and double-strand breaks. Progressive accumulation of DNA damage is an important mechanism in glioma carcinogenesis. On the other hand, as the mainstay in the treatment of glioma, radio- and chemotherapy exhibit their anticancer activity mainly via promoting DNA damages.

DNA double-strand breaks are the most perilous and lethal type of DNA damage, requiring timely repair [2]. Cells are estimated to suffer up to 50 DNA double-strand break events per day. Homologous recombination repair is one of the most important pathways to repair DNA double-strand breaks [2]. RAD51 protein family plays a critical role in homologous recombination-mediated DNA double-strand breaks repair. As a member of the RAD51 protein family, RAD51B can form a complex with other RAD51 paralogs, and then promote RAD51-mediated homologous DNA pairing and strand exchange [3]. Accumulative evidence has revealed that abnormal RAD51B is involved in carcinogenesis. Overexpression of *RAD51B* was found to promote cell proliferation and aneuploidy in gastric cancer [4]. Moreover, a high prevalence of *RAD51B* copy number alterations was observed in uterine leiomyosarcoma [5]. In addition, as an important DNA repair gene, *RAD51B* also has been identified to be associated with therapy resistance and prognosis of several cancers [4, 6, 7].

Enhancers are an important regulatory element dispersed widely throughout the human genome. Proper enhancer activity is essential for coordinated dynamic gene transcription. Accumulative evidence suggests that inappropriate enhancer activity is involved in cancer development and progression [8, 9]. Several cancer susceptibility variants identified by genome-wide association studies were found to affect regulatory activity of enhancers [8]. Significantly, a genome-wide analysis of somatic noncoding mutation patterns in cancer has revealed that *RAD51B* harbors frequent noncoding mutations in promoter and enhancer regions [10]. Therefore, it is worthwhile to evaluate the role of enhancer variants of *RAD51B* in glioma development and progression.

In this study, we conducted a case–control study consisting of 402 glioma patients and 654 controls to investigate whether enhancer variants of *RAD51B* confer susceptibility and progression to glioma. Then, the functional relevance of enhancer variants of *RAD51B* was explored.

## Methods

### Study subjects

This study comprised 402 glioma patients and 654 cancer-free population controls. Patients were recruited from Fujian, Zhejiang, and surrounding provinces between January 2010 and July 2016. Controls were cancer-free individuals recruited in the same time period. All subjects were unrelated Southern Han Chinese (CHS). For each patient, the pathologic diagnosis of glioma was confirmed according to the 2007 WHO classification of tumors of the central nervous system at initial diagnosis and reclassified according to the 2016 WHO classification of tumors of the central nervous system in this study [11, 12]. The progression-free survival (PFS) time of glioma patients was calculated as the time between date of initial treatment and date of progressive disease or death. The patients lost to follow-up or without progression were censored at the time of the last tumor evaluation. The date of last follow-up was February 3, 2020. The clinical characteristics of study subjects are presented in Additional file 1. Written informed consent was obtained from all subjects and this study was approved by the Institutional Review Board of the First Affiliated Hospital, Fujian Medical University.

### Selection of candidate genetic variations

VISTA Enhancer Browser (https://enhancer.lbl.gov/) was used to explore potential enhancers of *RAD51B* [13]. UCSC Genome Browser (http://www.genome.ucsc.edu/, GRCh37/hg19) was used to display the details of regions of interest [14]. The ENCODE regulation tracks show the H3K4Me1, H3K4Me3, and H3K27Ac histone modifications which are suggestive of enhancer and promoter. The DNase Clusters track displays regions where the chromatin is hypersensitive to DNase enzyme [15]. The ORegAnno track displays literature-curated regulatory elements [16]. In this study, we picked out the common enhancer variants with minor allelic frequency (MAF) of ≥ 10% in CHS population to assess the linkage disequilibrium (LD) status using Haploview v4.2 software. Then, tag variants were selected for genotyping.

### Genotype analysis

Genomic DNA samples were isolated from peripheral blood lymphocytes using Tiangen TIANamp Genomic DNA kit (Tiangen Biotech., Beijing, China). Genotype analysis was performed by Sequenom MassARRAY iPLEX platform (Sequenom Inc., San Diego, CA, USA). The representative clustering graph was presented in Additional file 2. We implemented several measures to control genotyping quality, including (1) genotyping was carried out without knowledge of the case/control status, (2) each assay plate contained both case and control samples, (3) each assay plate contained both positive and negative controls.

### Luciferase reporter gene assays

A 2759-bp DNA fragment corresponding to the enhancer hs1474 of *RAD51B* was subcloned into the pGL3-promoter vector (Promega, Madison, WI, USA). The resultant construct containing rs6573816 G allele was designated as P-G, while the other one containing rs6573816 C allele was designated as P-C. The constructs were sequenced to confirm their authenticity. Then, the constructs P-G, P-C, or the empty pGL3-promoter vector was co-electrotransfected with pRL-TK (Promega) into U251 cells, respectively. The luciferase activity was detected with a Dual-Luciferase Reporter Assay System (Promega). The U251 cells obtained from X–Y Biotechnology Corporation (Shanghai, China) were authenticated by DNA finger printing analysis and tested for mycoplasma contamination.

### Chromatin immunoprecipitation assays

Web application AliBaba2.1 (http://gene-regulation.com/pub/programs/alibaba2/index.html) was used to explore the transcription factor binding to rs6573816 locus with minimum matrix conservation of 70%. Then, chromatin immunoprecipitation (ChIP) assays were performed in U251 cells to verify the prediction. Cells were crosslinked in 1% formaldehyde followed by lysing and sonicating. Immunoprecipitation was performed using a BersinBio ChIP Kit (BersinBio Biotech., Guangzhou, Guangdong, China) and antibodies against predicted transcription factor POU2F1 or nonspecific rabbit IgG (Proteintech., Rosemont, IL, USA). Purified DNA was analyzed by PCR. Furthermore, to rule out nonspecific antibody-DNA interaction, we also analyzed the randomly selected region of *GAPDH* promoter by PCR. The PCR primers are presented in Additional file 3.

### siRNA mediated gene knockdown

We designed three silencing RNAs (siRNAs) to knockdown *POU2F1* expression. The *POU2F1* siRNAs and control siRNA were transfected into U251 cells respectively. The cells were collected after 48 h. Trizol (Life Technologies, Carlsbad, CA, USA) was used to isolate total RNA. RT-qPCR was used for analyses of *POU2F1* and *RAD51B* mRNA levels with TB Green Premix Ex Taq (Takara Bio., Shiga, Japan). The siRNAs and primers are summarized in Additional file 3. Western blot analyses were performed with primary antibodies against POU2F1 or RAD51B (Proteintech.) to measure protein levels. GAPDH was applied as a reference.

### Correlation analysis between rs6573816 and *RAD51B* expression

Gene Expression Omnibus (GEO, http://www.ncbi.nlm.nih.gov/geo/) was used to extract the expression data of *RAD51B* [17]. The dataset with GEO accession number GSE6536, which includes whole-genome gene expression data of the lymphoblastic cell lines from HapMap individuals, was used in this study [18]. The target ID of *RAD51B* is GI_19924114-I. And the gene expression data normalized by a median normalization method across all HapMap individuals was extracted. The genotype data of rs6573816 for the HapMap individuals was extracted from the International HapMap Project [19].

### Correlation analysis between the expression of *POU2F1 *and *RAD51B*

The normalized gene expression data of *POU2F1* and *RAD51B* measured in the lymphoblastic cell lines was extracted as described in the previous section. The target ID of *POU2F1* is GI_42476163-S. Meanwhile, the gene expression data from glioma tissues was extracted from the Chinese Glioma Genome Atlas (CGGA) database (http://www.cgga.org.cn/index.jsp) [20]. In this study, we used the dataset mRNAseq_693 which includes mRNA sequencing data from 693 glioma tissues.

### Statistical analysis

The associations between genotypes and glioma risk were estimated by logistic regression models. PFS curve was plotted using the Kaplan–Meier method, followed by log-rank test. Multivariable Cox regression with adjustment for sex, age, WHO grade, resection extent, radio- and chemotherapy, when appropriate, was performed to calculate hazard ratios (HRs) and 95% confidence intervals (CIs). The differences in luciferase reporter gene expression and gene expression levels were examined by a t-test. Pearson correlation was performed to analyze the expression correlation of *POU2F1* and *RAD51B*. Statistical analysis was carried out using Statistic Analysis System software (version 9.4, SAS Institute, Cary, NC, USA). The *P* value of < 0.05 was used as the criterion of statistical significance.

## Results

Four potential enhancers of *RAD51B* were identified by VISTA Enhancer Browser (hs1474, hs1392, hs1616, and hs1688). In silico analysis revealed that the degrees of enrichment of H3K4Me1 and H3K27Ac histone modifications in hs1474 are much higher than others (Additional file 4). More literature-curated regulatory elements were also found in hs1474 by ORegAnno track. Moreover, there are several DNaseI hypersensitive sites locating in hs1474. Therefore, we just picked out the common variants of hs1474 for further analyses. After linkage disequilibrium analysis, a common variant rs6573816 whose MAF is of 10% in CHS population was identified as tag variant for genotyping.

The results from the genotyping assay were shown in Table 1. The genotype frequencies of rs6573816 were 87.94% (GG genotype), 11.31% (GC genotype), 0.75% (CC genotype) in cases and 83.20% (GG), 15.87% (GC), and 0.93% (CC) in controls which were in agreement with that expected under Hardy–Weinberg equilibrium. Logistic regression showed that rs6573816 GC genotype was associated with a significantly decreased risk of glioma (odds ratio [OR] = 0.66, 95% CI 0.45–0.97; *P* = 0.034) compared with rs6573816 GG genotype. No significant association was found for rs6573816 CC genotype (*P* = 0.964), which might be due to the low frequency of rs6573816 CC genotype. When rs6573816 GC and CC genotypes were pooled for analysis, a significantly decreased risk of glioma was also found (OR = 0.68, 95% CI 0.47–0.99; *P* = 0.041).

**Table 1:** Genotype frequencies of rs6573816 and its association with glioma risk

Genotype^a^	Patients (n = 402) No. (%)	Controls (n = 654) No. (%)	OR^b^ (95%CI)	*P*
GG	350 (87.94)	540 (83.20)	1.00 (reference)	
GC	45 (11.31)	103 (15.87)	0.66 (0.45–0.97)	0.034
CC	3 (0.75)	6 (0.93)	1.03 (0.25–4.22)	0.964
GC + CC vs. GG			0.68 (0.47–0.99)	0.041

*OR* odds ratios, *CI* confidence interval

^a^Some samples failed to genotype

^b^Data were calculated by logistic regression with adjustment for age and sex

**Fig. 1:**
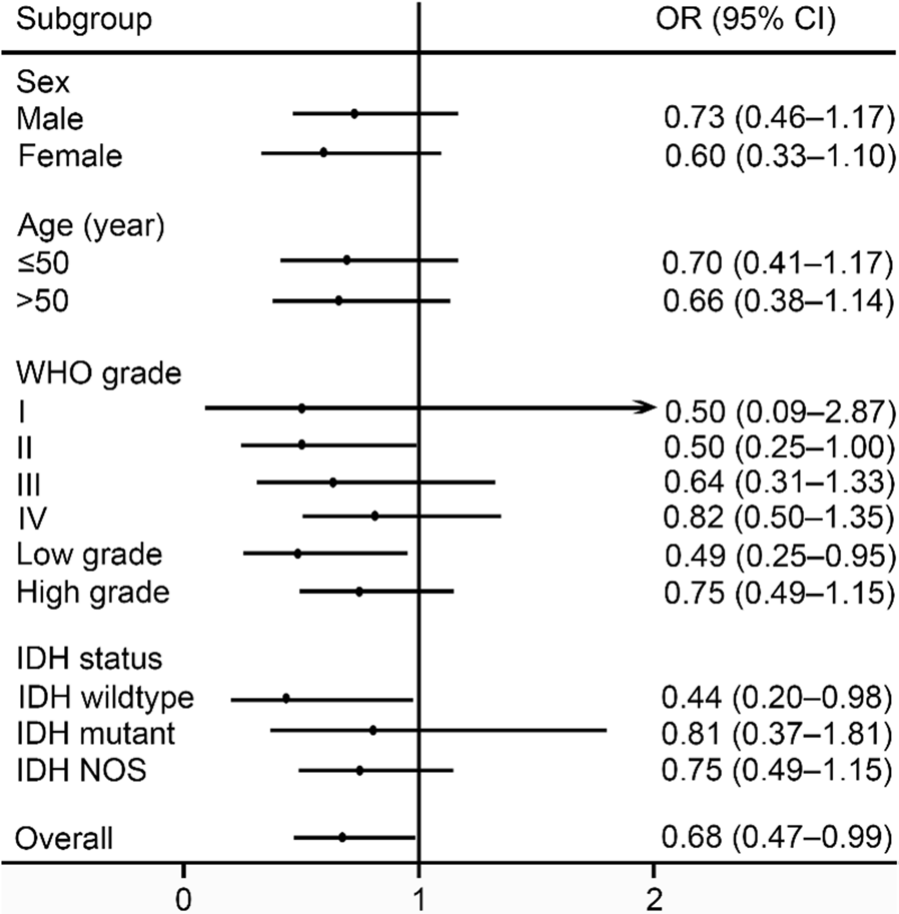
Subgroup analyses for rs6573816 based on sex, age, WHO grade, and *IDH* mutation status in a dominant model. Central black dots indicate ORs. Horizontal lines represent 95% CIs

Then, subgroup analyses for rs6573816 based on sex, age, WHO grade, and *IDH* mutation status were performed in a dominant model (Fig. 1). Downward trend in glioma risk of subjects with rs6573816 GC or CC genotype was observed in all subgroups. In low grade glioma and *IDH* wildtype glioma subgroups, significantly reduced risk of glioma was observed with ORs of 0.49 (95% CI 0.25–0.95; *P* = 0.034) and 0.44 (95% CI 0.20–0.98; *P* = 0.046) respectively. The relatively small sample size within a given stratum may be part of the reason why there was no significant association in other subgroups.

**Fig. 2:**
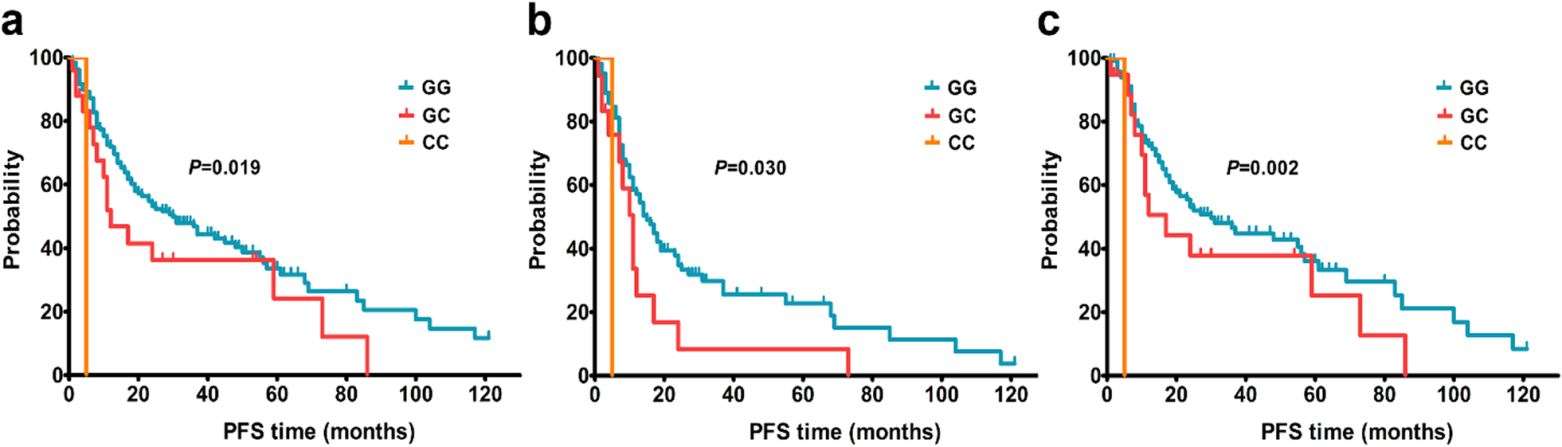
Kaplan–Meier estimates of PFS of the male glioma patients according to rs6573816 genotypes. **a** All male patients. **b** The male patients with high grade glioma. **c** The male patients underwent radiotherapy

The impact of clinical factors and rs6573816 on glioma progression was analyzed by log-rank test. The results indicated that age, WHO grade, and resection extent were significantly associated with glioma progression (Additional file 5). PFS was significantly improved in patients aged ≤ 50 years (*P* < 0.001), with lower grade glioma (*P* < 0.001), and underwent a more extensive resection (*P* = 0.002). The sex, radiotherapy, chemotherapy, and rs6573816 were not found to correlate with glioma progression in the whole group of patients (*P* > 0.05). Further subgroup analyses were employed to assess the impact of rs6573816 on glioma progression in the different groups of patients. As shown in Additional file 6 and Fig. 2a, rs6573816 was found to be significantly associated with PFS in the male glioma patients by log-rank test (*P* = 0.019). Multivariable Cox regression revealed that the association between rs6573816 and PFS in the male glioma patients just reach marginal significance level in a dominant model (Table 2, HR = 1.83, 95% CI 0.97–3.45; *P* = 0.061). However, further analyses indicated that rs6573816 was significantly associated with PFS in the male patients with high grade glioma (Fig. 2b, *P* = 0.030) or underwent radiotherapy (Fig. 2c, *P*  = 0.002). Multivariable Cox regression revealed that subjects with rs6573816 GC genotype had a worse PFS in the male patients with high grade glioma (Table 2, HR = 2.15, 95% CI 1.05–4.40; *P* = 0.036). When rs6573816 GC and CC genotypes were pooled for analysis, a worse PFS was also found (HR = 2.28, 95% CI 1.14–4.57; *P* = 0.020). In the male patients underwent radiotherapy, subjects with rs6573816 CC genotype had a worse PFS (HR = 8.76, 95% CI 1.01–76.11; *P* = 0.049). Interestingly, no significant association was found in the female glioma patients (Additional file 7).

**Table 2:** Multivariate analysis of rs6573816 on glioma progression in the different groups of male patients

Subgroup	Genotype	Median PFS (95%CI, months)	HR^a^ (95% CI)	*P*
All	GG	30 (19–48)	1.00 (reference)	
	GC	12 (7–59)	1.74 (0.90–3.34)	0.097
	CC	5 (NC)	4.80 (0.61–37.50)	0.135
	GC + CC	11 (7–59)	1.83 (0.97–3.45)	0.061
High grade glioma	GG	14 (11–19)	1.00 (reference)	
	GC	11 (4–17)	2.15 (1.05–4.40)	0.036
	CC	5 (NC)	8.04 (0.96–67.13)	0.054
	GC + CC	10 (4–12)	2.28 (1.14–4.57)	0.020
Underwent radiotherapy	GG	29 (19–48)	1.00 (reference)	
	GC	11 (6–59)	1.48 (0.68–3.21)	0.318
	CC	5 (NC)	8.76 (1.01–76.11)	0.049
	GC + CC	11 (5–59)	1.63 (0.78–3.43)	0.200

*PFS* progression-free survival, *CI* confidence interval, *HR* hazard ratio

^a^Data were calculated by multivariate Cox regression with adjustment for age, WHO grade, resection extent, radio- and chemotherapy when appropriate

**Fig. 3:**
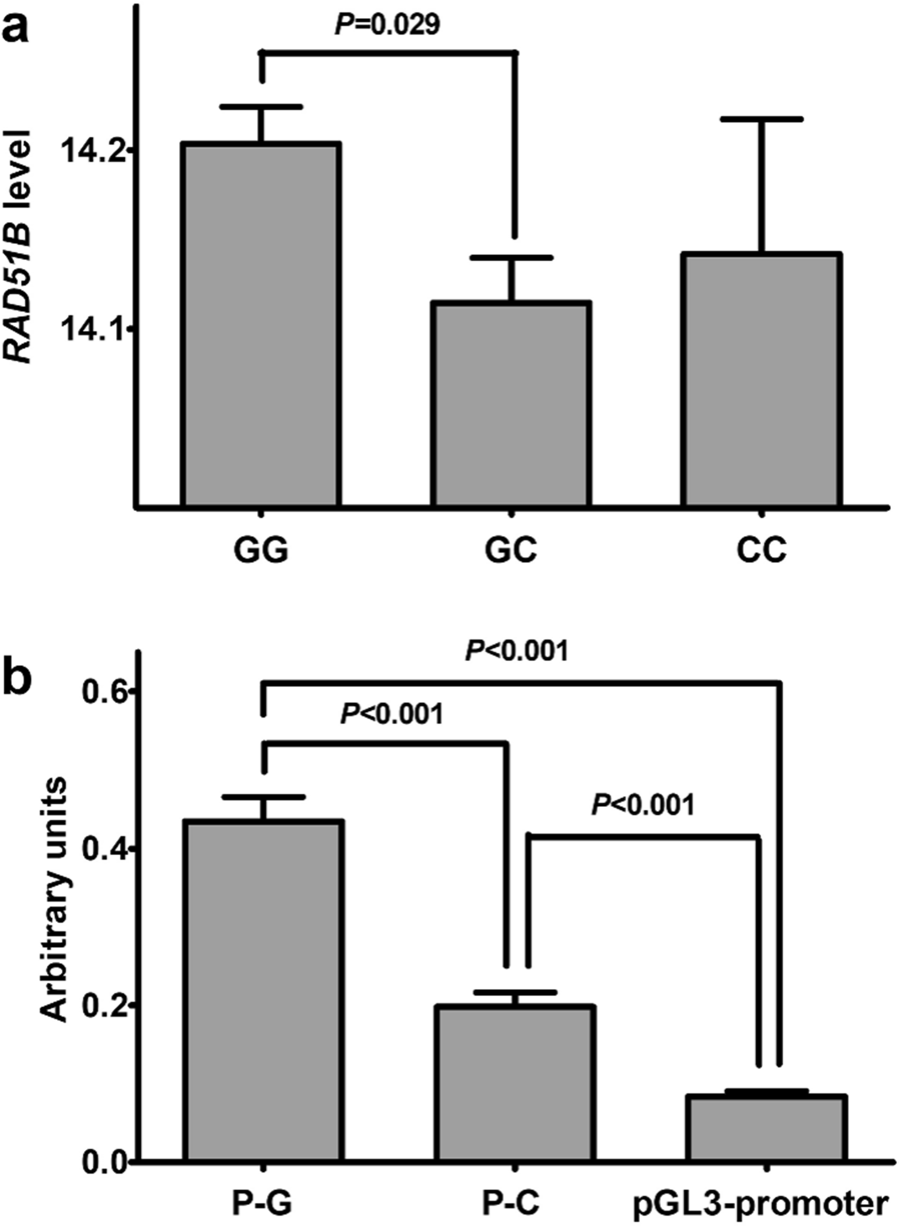
**a** The functional effect of rs6573816 on *RAD51B* expression in population (n = 270). **b** Reporter gene assays with constructs containing the potential enhancer (hs1474) with rs6573816 G allele (P-G) or C allele (P-C) in U251 cells. Columns represent means; Bars indicate SE

We further investigated whether rs6573816 has a functional effect on *RAD51B* expression in population. As shown in Fig. 3a, we found that subjects with rs6573816 GC genotype had lower expression levels of *RAD51B* than those with GG genotype (*P* = 0.029). To examine whether rs6573816 interferes with the regulatory activity of enhancer hs1474 in cell line, luciferase reporter gene assays were performed with constructs P-G and P-C encompassing hs1474 with rs6573816 G or C allele, respectively. As shown in Fig. 3b, both constructs P-G and P-C containing enhancer hs1474 drove significantly higher luciferase activity than the empty pGL3-promoter plasmid (both *P* < 0.001). Besides, significantly increased luciferase activity was observed for P-G compared with P-C (*P* < 0.001).

**Fig. 4:**
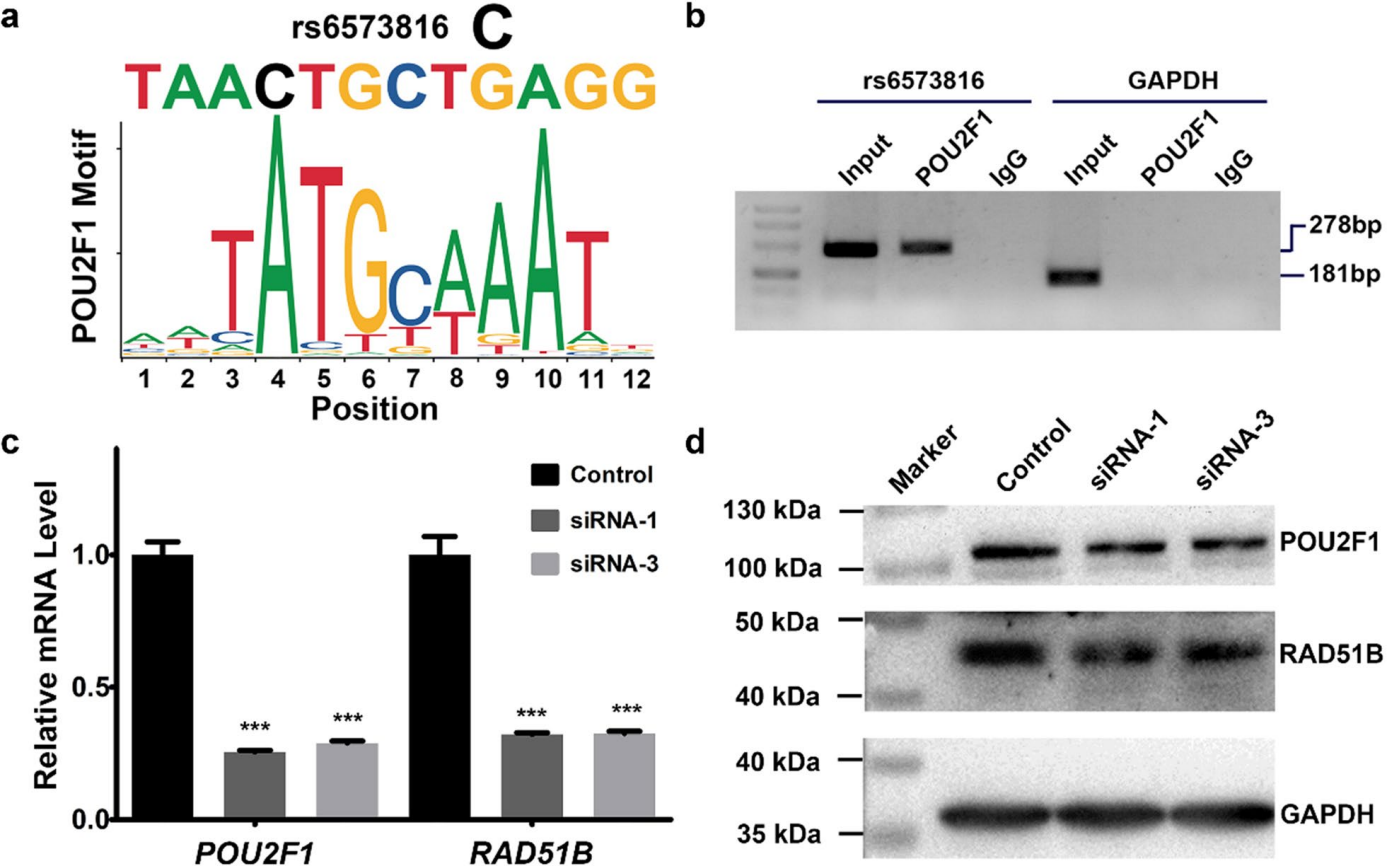
**a** rs6573816 resides within POU2F1 DNA-binding motif. **b** ChIP assays in U251 cells with rs6573816 GG genotype. The presence of POU2F1-binding *RAD51B* enhancer hs1474 was verified by PCR. **c** Depletion of *POU2F1* by RNAi attenuates the mRNA levels of *RAD51B*. Columns represent means; Bars indicate SE. ^***^ *P* < 0.001. **d** Knockdown of *POU2F1* by RNAi downregulates the protein levels of *RAD51B*

As shown in Fig. 4a, rs6573816 was predicted to be within the binding site of transcription factor POU2F1. In order to verify the prediction in cell line, we carried out ChIP assays in U251 cells with rs6573816 GG genotype. As shown in Fig. 4b, POU2F1 antibody immunoprecipitated rs6573816 locus specifically but not nonspecific region of *GAPDH* promoter. The results revealed the existence of POU2F1 binding to rs6573816 locus in cell line.

Then, siRNAs were designed to silence *POU2F1* expression to evaluate the effect of POU2F1 on *RAD51B* expression. The interference efficiency of three siRNAs was shown in Additional file 8. The siRNA-1 and siRNA-3 with higher interference efficiency were chosen for further analysis. As shown in Fig. 4c, the siRNA-1 and siRNA-3 significantly reduced the relative mRNA levels of *POU2F1* (both *P* < 0.001). Follow by, the relative mRNA levels of *RAD51B* were also significantly decreased (both *P* < 0.001). Consistent with the mRNA trend, the siRNA-1 and siRNA-3 significantly downregulated the protein expression of POU2F1, which was accompanied by attenuated protein expression of RAD51B (Fig. 4d).

**Fig. 5:**
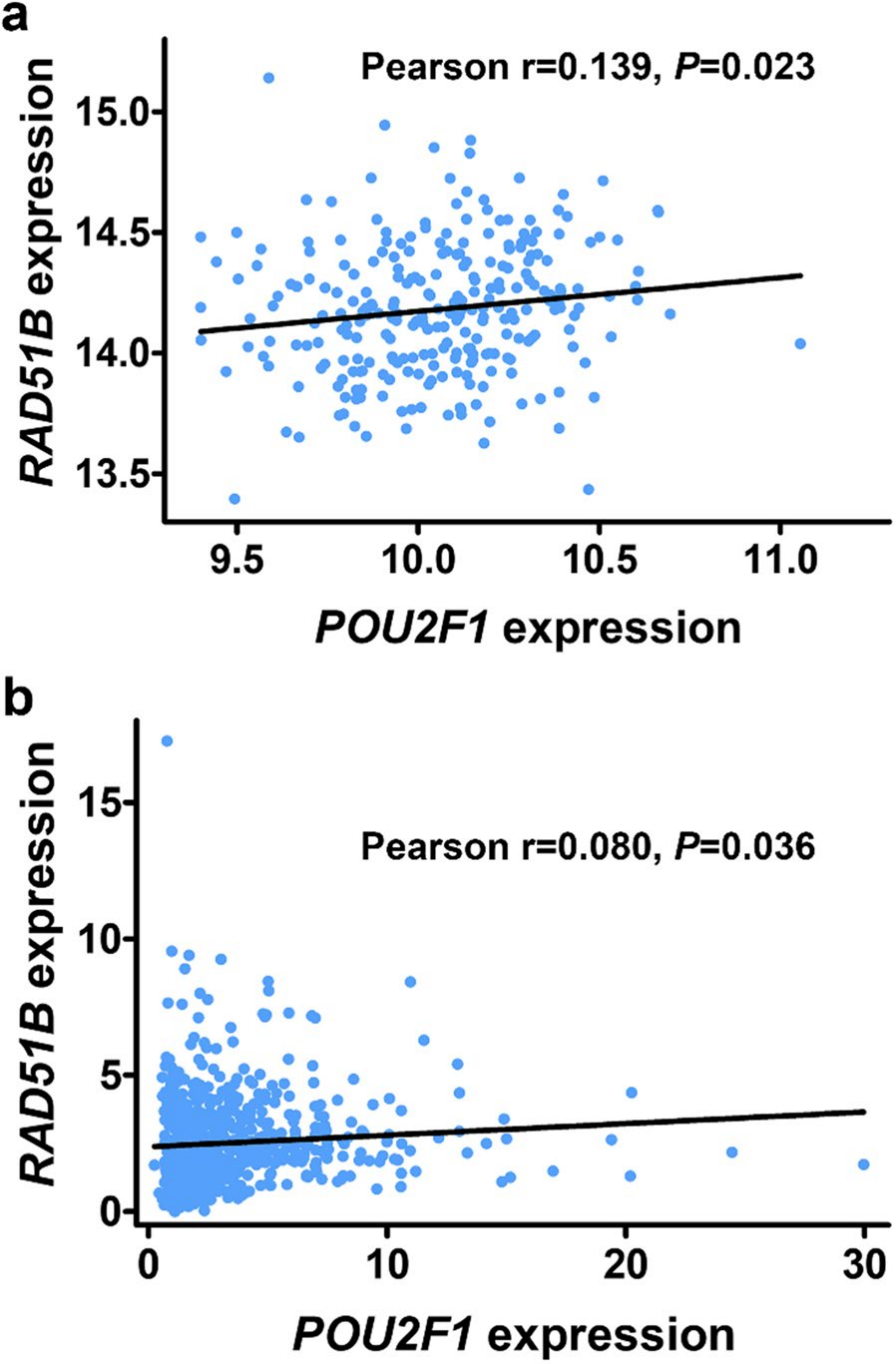
Correlation between the expression of *POU2F1* and *RAD51B* in lymphoblastic cells and glioma tissues. **a** Lymphoblastic cells from HapMap individuals (n = 270). **b** Glioma tissues from CGGA database (n = 693)

We further explored the relationship between the expression of *POU2F1* and *RAD51B* in lymphoblastic cells and glioma tissues. In lymphoblastic cells, positive correlation was observed between the expression of *POU2F1* and *RAD51B*, with Pearson r value being 0.139 (*P* = 0.023, Fig. 5a). Consistently, positive correlation was also observed in glioma tissues, with Pearson r value being 0.080 (*P* = 0.036, Fig. 5b).

## Discussion

In the present study, an enhancer variant of *RAD51B* rs6573816 was found to be significantly associated with glioma susceptibility and progression in a sex-dependent manner. In addition, we found that rs6573816 C allele might downregulate *RAD51B* expression by affecting transcription factor POU2F1 binding.

The DNA double-strand break is a particularly deleterious form of DNA damage induced by ionizing radiation [2]. Unrepaired and misrepaired DNA double-strand breaks contribute to the genomic instability, and then lead to cancer. Therefore, it is not surprising that the imbalance of DNA double-strand break repair pathways results in accumulation of deleterious DNA damage and is associated with carcinogenesis. Previous studies have revealed that genetic lesions disrupting DNA double-strand break repair pathways are involved in carcinogenesis [21]. RAD51 protein family is essential for DNA double-strand break repair by homologous recombination. In the present study, we found that an enhancer variant of *RAD51B* rs6573816 was associated with glioma susceptibility, which implies a significant role of *RAD51B* in glioma development. Of note, the rs6573816 C allele driving lower expression of *RAD51B* was found to decrease the risk of glioma significantly. Logically, our findings indicate that overexpression of *RAD51B* might be a risk factor for glioma. Interestingly, an intron variant of *RAD51B* rs2189517 has been demonstrated to be associated with rectal cancer risk [22]. We investigated the functional effect of rs2189517 on *RAD51B* expression in lymphoblastic cells from HapMap individuals as mentioned above. In consistent with our results, the risk rs2189517 G allele was significantly associated with higher expression of *RAD51B* (Additional file 9). Furthermore, upregulated expression of *RAD51B* was observed in gastric precancer and cancer tissues. It has been revealed that overexpression of *RAD51B* leads to enhanced cell proliferation and elevated genetic instability [4]. Likewise, increased protein level of RAD51, another member of the RAD51 protein family, has been observed in various cancers [23]. In myeloma and Barrett’s esophageal adenocarcinoma, overexpression of *RAD51* was found to enhance homologous recombination activity and then lead to pathological recombination events [24, 25]. These results suggest that the precisely regulated activity of homologous recombination is critical for the maintenance of genome stability. While, overexpression of *RAD51B* might function as a susceptibility factor for glioma.

DNA repair is considered as a double-edged sword because of its diversified roles in cancer initiation, progression, and therapy. For instance, *ERCC1* variant rs2298881 G allele increasing the risk of lung cancer development is associated with better prognosis [26]. In the present study, we also found that the rs6573816 C allele, which was identified as a protective allele for glioma development and drove lower expression of *RAD51B*, increased the risk of glioma progression in a sex-dependent manner. These results indicate that low expression of *RAD51B* might be an adverse prognostic factor for glioma. Consistently, high expression of *RAD51B* has been found to be associated with a favorable prognosis for breast cancer and lung cancer [6, 7]. Previous study has demonstrated that overexpression of *RAD51B* can induce cell apoptosis after irradiation [27]. On the other hand, sex differences in glioma in terms of incidence, prognosis, pathophysiology, and cancer biology have been widespread concern [28]. Our previous studies also found that hematologic and serum biomarkers are sex-dependently associated with glioma grade and prognosis [29, 30]. This study provides additional evidence that there may be sex-specific effects of genetic variations on glioma progression.

## Conclusions

In conclusion, an enhancer variant of *RAD51B* rs6573816 was identified as a novel susceptibility and progression locus for glioma. And subjects with rs6573816 GC or CC genotype experienced a downward trend in glioma risk in all subgroups. While the rs6573816 C allele increased the risk of disease progression in a sex-dependent manner. The rs6573816 C allele was found to downregulate the expression of *RAD51B* by disrupting a POU2F1 binding site. These results indicate that an enhancer variant of *RAD51B* rs6573816 influences enhancer activity by changing a POU2F1 binding site and confers susceptibility and progression to glioma.

### Supplementary Information

**Additional file 1: Table S1.** Clinical characteristics of glioma patients and controls.

**Additional file 2: Figure S1.** The representative clustering graph of rs6573816 genotyping.

**Additional file 3: Table S2.** Oligonucleotides for ChIP-PCR, RNAi, and RT-qPCR.

**Additional file 4: Figure S2.** In silico analysis of four potential enhancers of *RAD51B*.

**Additional file 5: Table S3.** Univariate analysis of clinical factors and rs6573816 on glioma progression.

**Additional file 6: Table S4.** Univariate subgroup analysis of rs6573816 on glioma progression.

**Additional file 7: Figure S3.** Kaplan–Meier estimates of PFS of the female glioma patients according to rs6573816 genotypes. (A) All female patients. (B) The female patients with high grade glioma. (C) The female patients underwent radiotherapy.

**Additional file 8: Figure S4.** Interference efficiency of the candidate siRNA oligonucleotides for *POU2F1*. (A) The mRNA levels of *POU2F1* after transfecting siRNAs. Columns represent means; Bars indicate SE. (B) The protein levels of *POU2F1* after transfecting siRNAs.

**Additional file 9: Figure S5.** The functional effect of rs2189517 on *RAD51B* expression in population (n = 270).

## Data Availability

The datasets used and/or analyzed during the current study are available from the corresponding author on reasonable request.

## Abbreviations

CHS: Southern Han Chinese
PFS: Progression-free survival
MAF: Minor allelic frequency
LD: Linkage disequilibrium
ChIP: Chromatin immunoprecipitation
siRNA: Silencing RNA
GEO: Gene Expression Omnibus
CGGA: Chinese Glioma Genome Atlas
HR: Hazard ratio
CI: Confidence interval
OR: Odds ratios
