# Supplementary material for: Identification of a RAD51B enhancer variant for susceptibility and progression to glioma
Source: Cancer Cell Int. 2023 Oct 19;23:246. doi: 10.1186/s12935-023-03100-8 (PMC10585866; doi:10.1186/s12935-023-03100-8)
Supplement: Supplementary file 1 — Additional file 1: Table S1. Clinical characteristics of glioma patients and controls. [file 12935_2023_3100_MOESM1_ESM.docx]

**Table S1.** Clinical characteristics of glioma patients and controls.

| Characteristics | Patients (n=402) | Controls (n=654) |
| --- | --- | --- |
| Age, mean (SD, years) | 45.44 (15.22) | 50.72 (11.31) |
| Sex, n (%) |  |  |
| Male | 243 (60.45) | 413 (63.15) |
| Female | 159 (39.55) | 241 (36.85) |
| WHO grade, n (%) |  |  |
| I | 24 (5.97) |  |
| II | 118 (29.35) |  |
| III | 81 (20.15) |  |
| IV | 158 (39.30) |  |
| Unascertainable | 21 (5.23) |  |
| Pathology, n (%) |  |  |
| Diffuse astrocytoma, IDH-mutant | 15 (3.73) |  |
| Diffuse astrocytoma, IDH-wildtype | 3 (0.75) |  |
| Diffuse astrocytoma, NOS | 36 (8.96) |  |
| Anaplastic astrocytoma, IDH-mutant | 6 (1.49) |  |
| Anaplastic astrocytoma, IDH-wildtype | 5 (1.24) |  |
| Anaplastic astrocytoma, NOS | 21 (5.22) |  |
| Glioblastoma, IDH-wildtype | 72 (17.91) |  |
| Glioblastoma, IDH-mutant | 7 (1.74) |  |
| Glioblastoma, NOS | 79 (19.65) |  |
| Oligodendroglioma, IDH-mutant and 1p/19q-codeleted | 14 (3.48) |  |
| Oligodendroglioma, NOS | 33 (8.21) |  |
| Anaplastic oligodendroglioma, IDH-mutant and 1p/19q-codeleted | 11 (2.74) |  |
| Anaplastic oligodendroglioma, NOS | 23 (5.72) |  |
| Others and nonclassification | 77 (19.16) |  |
| Resection extent, n (%) |  |  |
| Gross total resection | 101 (25.12) |  |
| Partial resection | 123 (30.60) |  |
| Biopsy | 6 (1.49) |  |
| Unascertainable | 172 (42.79) |  |
| Radiotherapy, n (%) | 225 (55.97) |  |
| Chemotherapy, n (%) | 278 (69.15) |  |
