# Supplementary figures and images for: Identification of a RAD51B enhancer variant for susceptibility and progression to glioma
Source: Cancer Cell Int. 2023 Oct 19;23:246. doi: 10.1186/s12935-023-03100-8 (PMC10585866; doi:10.1186/s12935-023-03100-8)

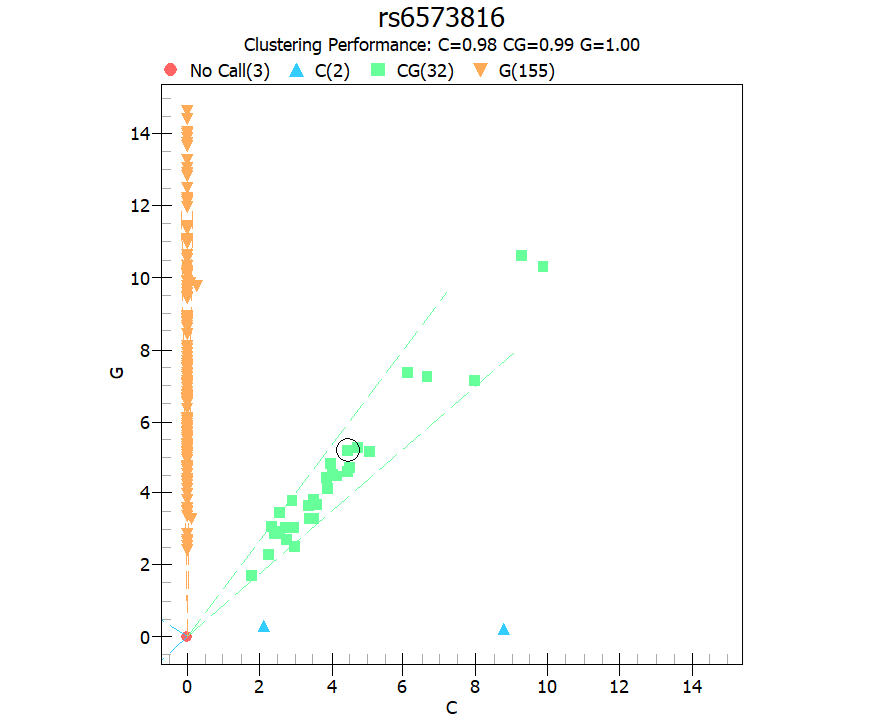

Supplement: Supplementary file 2 — Additional file 2: Figure S1. The representative clustering graph of rs6573816 genotyping. [file 12935_2023_3100_MOESM2_ESM.tif]

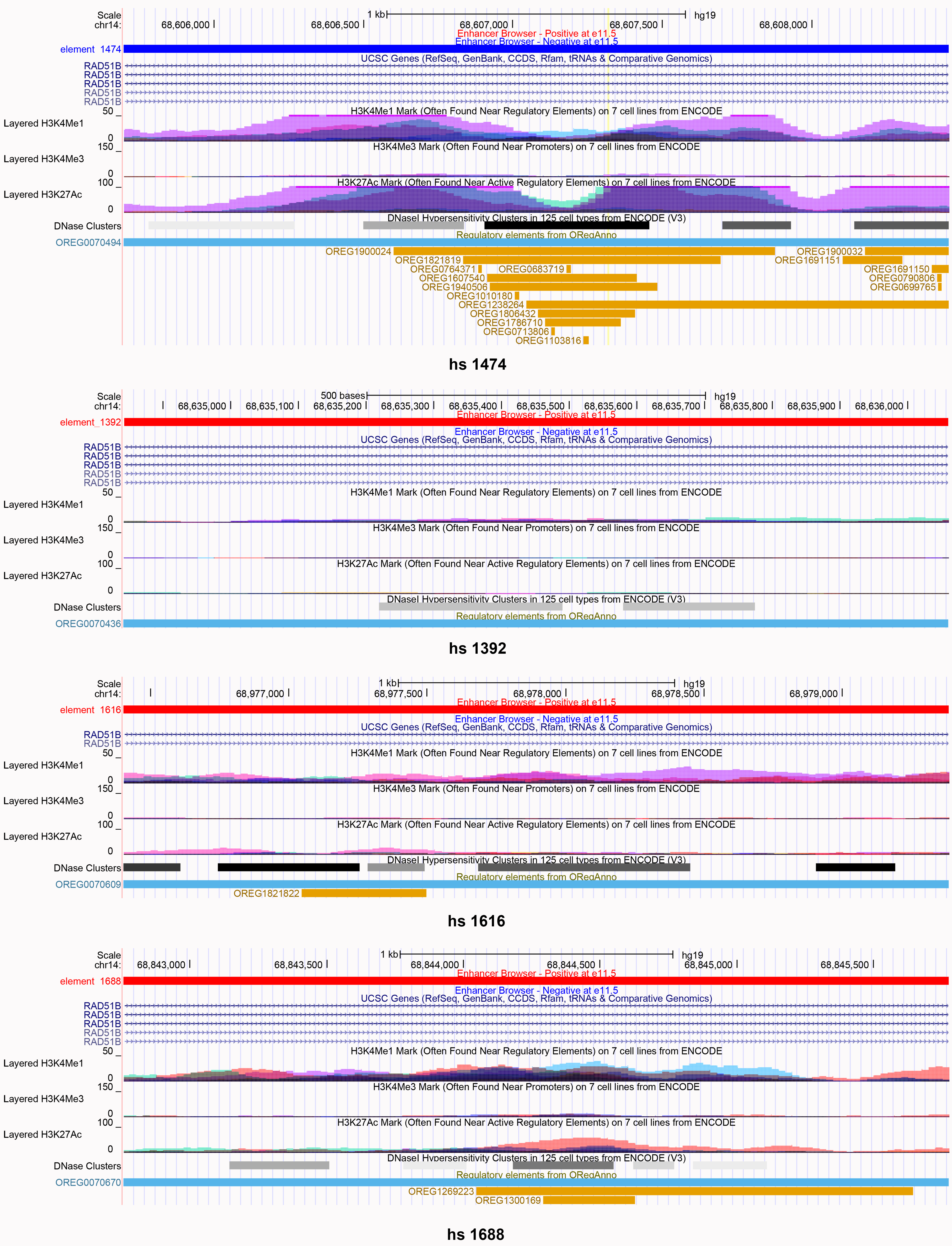

Supplement: Supplementary file 4 — Additional file 4: Figure S2. In silico analysis of four potential enhancers of RAD51B. [file 12935_2023_3100_MOESM4_ESM.tif]

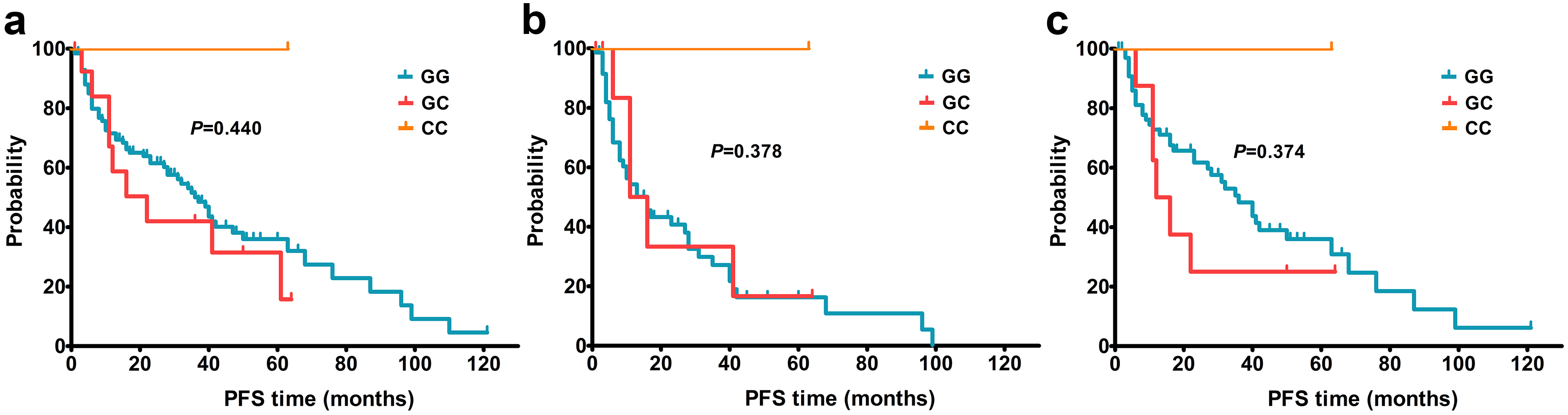

Supplement: Supplementary file 7 — Additional file 7: Figure S3. Kaplan–Meier estimates of PFS of the female glioma patients according to rs6573816 genotypes. (A) All female patients. (B) The female patients with high grade glioma. (C) The female patients underwent radiotherapy. [file 12935_2023_3100_MOESM7_ESM.tif]

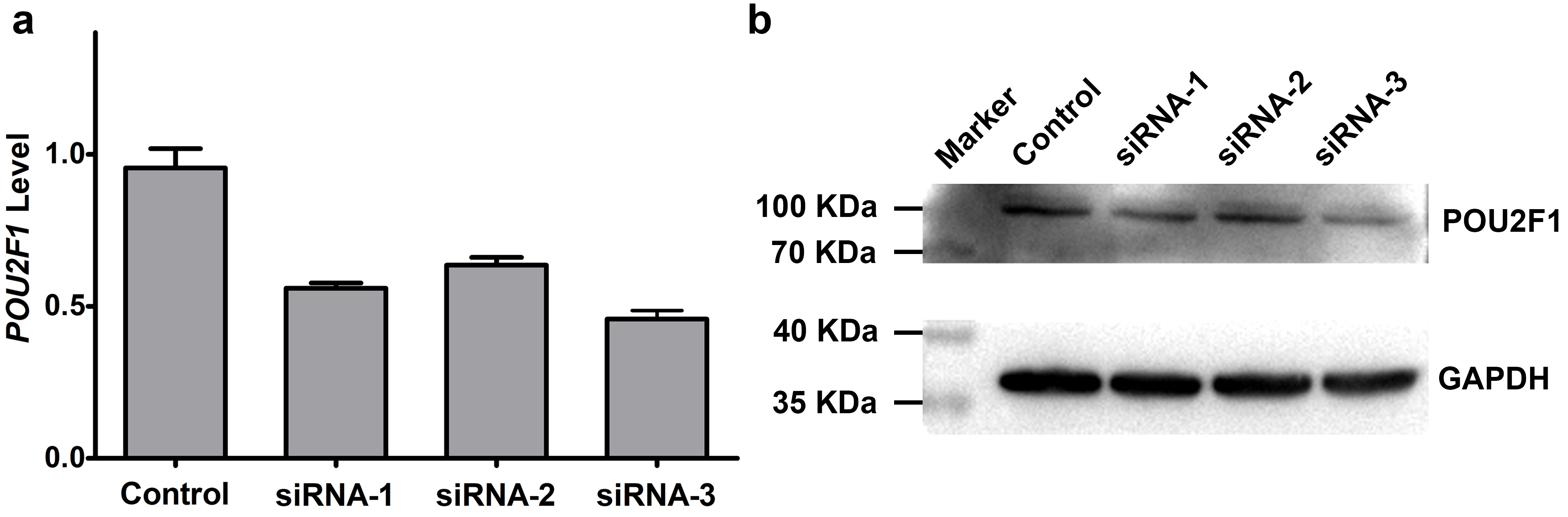

Supplement: Supplementary file 8 — Additional file 8: Figure S4. Interference efficiency of the candidate siRNA oligonucleotides for POU2F1. (A) The mRNA levels of POU2F1 after transfecting siRNAs. Columns represent means; Bars indicate SE. (B) The protein levels of POU2F1 after transfecting siRNAs. [file 12935_2023_3100_MOESM8_ESM.tif]

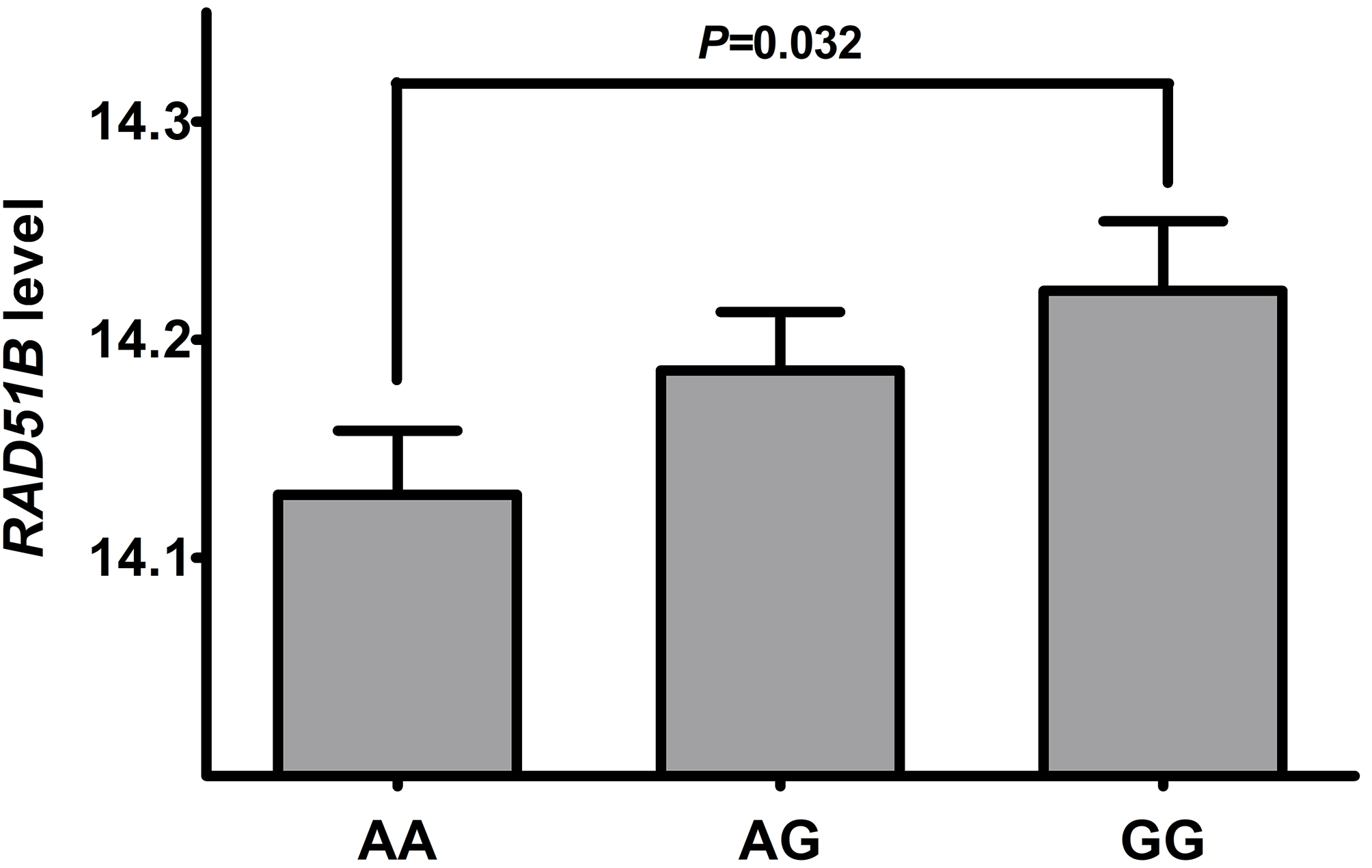

Supplement: Supplementary file 9 — Additional file 9: Figure S5. The functional effect of rs2189517 on RAD51B expression in population (n = 270). [file 12935_2023_3100_MOESM9_ESM.tif]
