# Supplementary material for: Identification of a RAD51B enhancer variant for susceptibility and progression to glioma
Source: Cancer Cell Int. 2023 Oct 19;23:246. doi: 10.1186/s12935-023-03100-8 (PMC10585866; doi:10.1186/s12935-023-03100-8)
Supplement: Supplementary file 3 — Additional file 3: Table S2. Oligonucleotides for ChIP-PCR, RNAi, and RT-qPCR. [file 12935_2023_3100_MOESM3_ESM.docx]

**Table S2.** Oligonucleotides for ChIP-PCR, RNAi, and RT-qPCR.

| Experiment | Designation | Sequence (5’-3’) |
| --- | --- | --- |
| ChIP | rs6573816-F | ACCCCTAACAACCACAGCTAC |
|  | rs6573816-R | CCTAACTCTGTGAGCATGGCA |
|  | GAPDH-F | AAAGTAGGGCCCGGCTACTA |
|  | GAPDH-R | TCGAACAGGAGGAGCAGAGA |
| RNAi | siRNA-1 | CCAGCAGCUCACCUAUUAA UU |
|  | siRNA-2 | GCAGCAGACUCAAGAAUGA UU |
|  | siRNA-3 | CCAGCAUAGAGACCAACAU UU |
|  | siRNA-Control | UUCUCCGAACGUGUCACGU UU |
| RT-qPCR | POU2F1-F | TGCAGCAACTACCCTCACAG |
|  | POU2F1-R | CGGTTGCTGTGTTGTTGGAG |
|  | RAD51B-F | ACAGTGTGAATACCCGGCTG |
|  | RAD51B-R | GAGCCCAGTTCAGTTCAGCT |
|  | GAPDH-F | AACGGATTTGGTCGTATTGGG |
|  | GAPDH-R | CCTGGAAGATGGTGATGGGAT |
|  |  |  |
