# Supplementary material for: Identification of a RAD51B enhancer variant for susceptibility and progression to glioma
Source: Cancer Cell Int. 2023 Oct 19;23:246. doi: 10.1186/s12935-023-03100-8 (PMC10585866; doi:10.1186/s12935-023-03100-8)
Supplement: Supplementary file 5 — Additional file 5: Table S3. Univariate analysis of clinical factors and rs6573816 on glioma progression. [file 12935_2023_3100_MOESM5_ESM.docx]

**Table S3.** Univariate analysis of clinical factors and rs6573816 on glioma progression.

| Variable | Median PFS (95%CI, months) | | *P*^†^ |
| --- | --- | --- | --- |
| Sex |  |  | 0.646 |
| Male |  | 25 (18–42) |  |
| Female |  | 37 (23–47) |  |
| Age (years) |  |  | <0.001 |
| ≤50 |  | 41 (36–56) |  |
| >50 |  | 13 (11–19) |  |
| WHO grade |  |  | <0.001 |
| I |  | 86 (11–100) |  |
| II |  | 61 (50–83) |  |
| III |  | 40 (23–55) |  |
| IV |  | 10 (8–12) |  |
| WHO grade |  |  | <0.001 |
| Low grade |  | 61 (50–83) |  |
| High grade |  | 14 (11–17) |  |
| Resection extent |  |  | 0.002 |
| Gross total resection |  | 55 (28–NC) |  |
| Partial resection |  | 16 (10–19) |  |
| Biopsy |  | 12 (NC) |  |
| Radiotherapy |  |  | 0.487 |
| Yes |  | 31 (22–41) |  |
| No |  | 37 (19–45) |  |
| Chemotherapy |  |  | 0.136 |
| Yes |  | 28 (19–37) |  |
| No |  | 42 (23–86) |  |
| rs6573816 |  |  | 0.268 |
| GG |  | 34 (24–41) |  |
| GC |  | 16 (11–59) |  |
| CC |  | NC |  |

^†^ Data were calculated by log-rank test.

PFS: progression free survival; CI: confidence interval; NC: not calculable.
