# Supplementary material for: Identification of a RAD51B enhancer variant for susceptibility and progression to glioma
Source: Cancer Cell Int. 2023 Oct 19;23:246. doi: 10.1186/s12935-023-03100-8 (PMC10585866; doi:10.1186/s12935-023-03100-8)
Supplement: Supplementary file 6 — Additional file 6: Table S4. Univariate subgroup analysis of rs6573816 on glioma progression. [file 12935_2023_3100_MOESM6_ESM.docx]

**Table S4.** Univariate subgroup analysis of rs6573816 on glioma progression.

| Subgroup | *P*^†^ |
| --- | --- |
| Sex |  |
| Male | 0.019 |
| Female | 0.440 |
| Age (years) |  |
| ≤50 | 0.221 |
| >50 | 0.640 |
| WHO grade |  |
| I | 0.366 |
| II | 0.865 |
| III | 0.176 |
| IV | 0.606 |
| WHO grade |  |
| Low grade | 0.722 |
| High grade | 0.334 |
| Resection extent |  |
| Gross total resection | 0.362 |
| Partial resection | 0.092 |
| Biopsy | 0.786 |
| Radiotherapy |  |
| Yes | 0.297 |
| No | 0.903 |
| Chemotherapy |  |
| Yes | 0.218 |
| No | 0.906 |

^†^ Data were calculated by log-rank test.
